# Association Between Cholecystectomy and Gastric Cancer Risk: A Systematic Review and Meta-Analysis

**DOI:** 10.3389/fonc.2022.667736

**Published:** 2022-01-31

**Authors:** Ying Yang, Ming-Hua Liu, Yan Li

**Affiliations:** Department of Gastroenterology, Shengjing Hospital of China Medical University, Shenyang, China

**Keywords:** cholecystectomy, gastric cancer, meta-analysis, observational study, systematic review

## Abstract

**Objectives:**

Although several epidemiological studies have attempted to evaluate the relationship between cholecystectomy and gastric cancer risk, the findings have been controversial. This study aimed to carry out a systematic review and meta-analysis following the reporting guidelines to comprehensively analyze and quantify the evidence of the aforementioned association.

**Methods:**

Studies were identified by searching the Medline (PubMed), Embase, and Web of Science from inception to November 30, 2020, with only studies published in English being considered. Summary relative risks (RRs) and 95% confidence intervals (CIs) were calculated by random-effects models.

**Results:**

Eight studies (five cohort studies and three case–control studies) with a total of 26,063 gastric cancer patients and 848,081 participants were included. The summarized RR of the relationship between cholecystectomy and gastric cancer risk was 1.11 (95%CI: 1.03–1.20), with low heterogeneity (*P* = 0.117, *I* ^2^ = 37.8%). These positive findings were consistent in most subgroup analyses like region in Asia, number of cases ≥200, cohort study design, sex in male, low risk of bias, exposure collection by database, and adjustments made for age, gender, calendar year. Of note, we also observed positive association between cholecystectomy and non-cardia of gastric cancer risk (RR = 1.17, 95%CI: 1.04–1.33). No publication bias was present.

**Conclusions:**

The aforementioned evidence suggested that a history of cholecystectomy was associated with a slightly elevated risk of gastric cancer. Results of most subgroup analyses also supported the main findings. More prospective studies are warranted to further validate these findings.

## Introduction

Gastric cancer (GC) is one of the most common cancers in the world. Although the incidence of GC is decreasing, it is still the sixth most common malignancy and the third leading cause of cancer-related deaths. The incidence of GC is significantly elevated in East Asia, while that in North America, northern Europe, and the entire African region is generally lower (1, 2). GC is a multifactorial disease with several risk factors, such as *Helicobacter pylori* infection, consumption of foods preserved by salting, low intake of fruits, alcohol consumption, and active tobacco smoking (3–5).

Cholelithiasis (i.e., the presence of gallstones) is the most common gastrointestinal disease. An estimated 10% of Europeans and Americans and 5–10% of Asians are carriers of gallbladder stones (6, 7). The incidence of cholelithiasis is still on the rise, with the improvement in living standards and the extension of life expectancy. Cholecystectomy, especially laparoscopic cholecystectomy, is the standard therapy for uncomplicated gallstone disease (8). Although cholecystectomy can improve inflammation, it may also increase duodenal gastric reflux (9), which has been proposed to increase the risk of several types of cancers in digestive system organs, such as liver cancer, colorectal cancer, and pancreatic cancer (10–12). Recent epidemiological evidence investigating the relationship between cholecystectomy and GC risk has been reported. In 2012, Ge and colleagues (13) carried out a systematic review and meta-analysis that suggested that cholecystectomy did not increase the overall risk of GC. However, some later published studies with a larger sample size generated different findings, which were different from the previous meta-analysis. For example, Chen et al. conducted a cohort study with 202 GC patients and 77,725 participants and observed that cholecystectomy was significantly associated with the risk of GC throughout the follow-up periods (14). Of note, the results of these published cohort studies have been conflicting, which might be attributed to the differences in the number of participants and the years of follow-up (14–17).

Considering that the previous meta-analysis is out of date and does not include research from the past decade, and given the conflicting conclusions of current research, this study aimed to carry out an updated systematic review and meta-analysis of observational studies to evaluate the association between cholecystectomy and the risk of GC.

## Materials and Methods

The reporting standards of the Meta-Analysis of Observational Studies in Epidemiology group (18) and Preferred Reporting Items for Systemic Reviews and Meta-Analyses (PRISMA) (**Supplementary Table Sl**) guidelines (19) for systematic reviews and meta-analyses of non-randomized controlled trials were followed in the present study.

### Search Strategy

Two independent individuals (YY and M-HL) comprehensively searched the Medline (PubMed), Embase, and Web of Science from inception to November 30, 2020, with only studies published in English being considered. Details of the full search strategy are provided in the **Supplementary Table S2**. Furthermore, the reference lists of all included studies and pertinent reviews and meta-analyses were manually examined to identify additional eligible studies.

### Study Selection

A study was eligible for inclusion if it (1) utilized an observational study design; (2) evaluated the relationship between cholecystectomy and GC risk; and (3) demonstrated estimates of odds ratios (ORs), relative risks (RRs), or hazard ratios (HRs) with 95% confidence intervals (CIs) or extractable data necessary to calculate these parameters. However, publications meeting any of the following criteria were excluded: (1) clinical trials, letters, editorials, case reports, reviews, meta-analyses, and meeting abstracts; (2) lack of sufficient risk estimates or related data to calculate risk estimates; and (3) not published in English. The selection and exclusion of studies were reviewed by two investigators (YY and M-HL). Disagreements were resolved by consensus with a third author (YL).

### Data Abstraction and Quality Assessment

Data were extracted in duplicate using standardized forms. Disagreements were resolved by consensus. The following information was collected: last name of the first author, publication year, geographical location, study design, number of cases, number of controls/cohorts, and characteristics of exposure and covariates matched in the study design or adjusted in the statistical analysis.

Quality assessment was performed using the Newcastle–Ottawa Scale (NOS), which consisted of eight items grouped into three domains (selection, comparability, and exposure/outcome) to assess the methodological quality of case–control or cohort studies (20). Studies that achieved a full rating in at least two categories of the three assessments were considered to have a low risk of bias (21).

### Statistical Analysis

The risk estimates were extracted from the original studies, namely, standardized incidence ratio, HR, OR, and RR. As the absolute risk of GC was low, the other risk estimates were considered similar estimates to RR (22). The random-effects model, which considers both within- and between-study variations, was used to summarize RR with their 95%CI of each study (23). Heterogeneity among studies was assessed with *I* ^2^ statistics. *I^2^;* estimates the proportion of variability in the meta-analysis caused by differences between studies instead of sampling error (24). The larger *I^2^* indicated the greater heterogeneity of the studies included in the meta-analysis. Meanwhile, *P*-values are generated according to the degree of heterogeneity in Forest plot (24). Cutoff points ≤25%, ≤50%, ≤75%, and >75% indicated no, low, moderate, and significant heterogeneities, respectively (24). Subgroup analyses were conducted to probe into heterogeneous sources by using pre-specified variables like region, anatomic subsite of GC, number of cases, study design, sex, risk of bias, exposure collection, and adjustments made for potential confounders, namely, age, sex, and calendar year. Associations that resulted from studies with small study biases (e.g., publication bias) were evaluated by visual inspection of funnel plot and formal testing using Egger’s test and Begg’s test (25, 26). Sensitivity analysis was conducted in which the summarized risk estimates were recalculated by omitting one study at a time so as to assess the effect of individual studies on the estimated RR (27). All statistical analyses were performed using Stata 12.0 software (Stata LLC, TX, USA).

## Results

### Summary of the Selection Process

**Figure 1:**
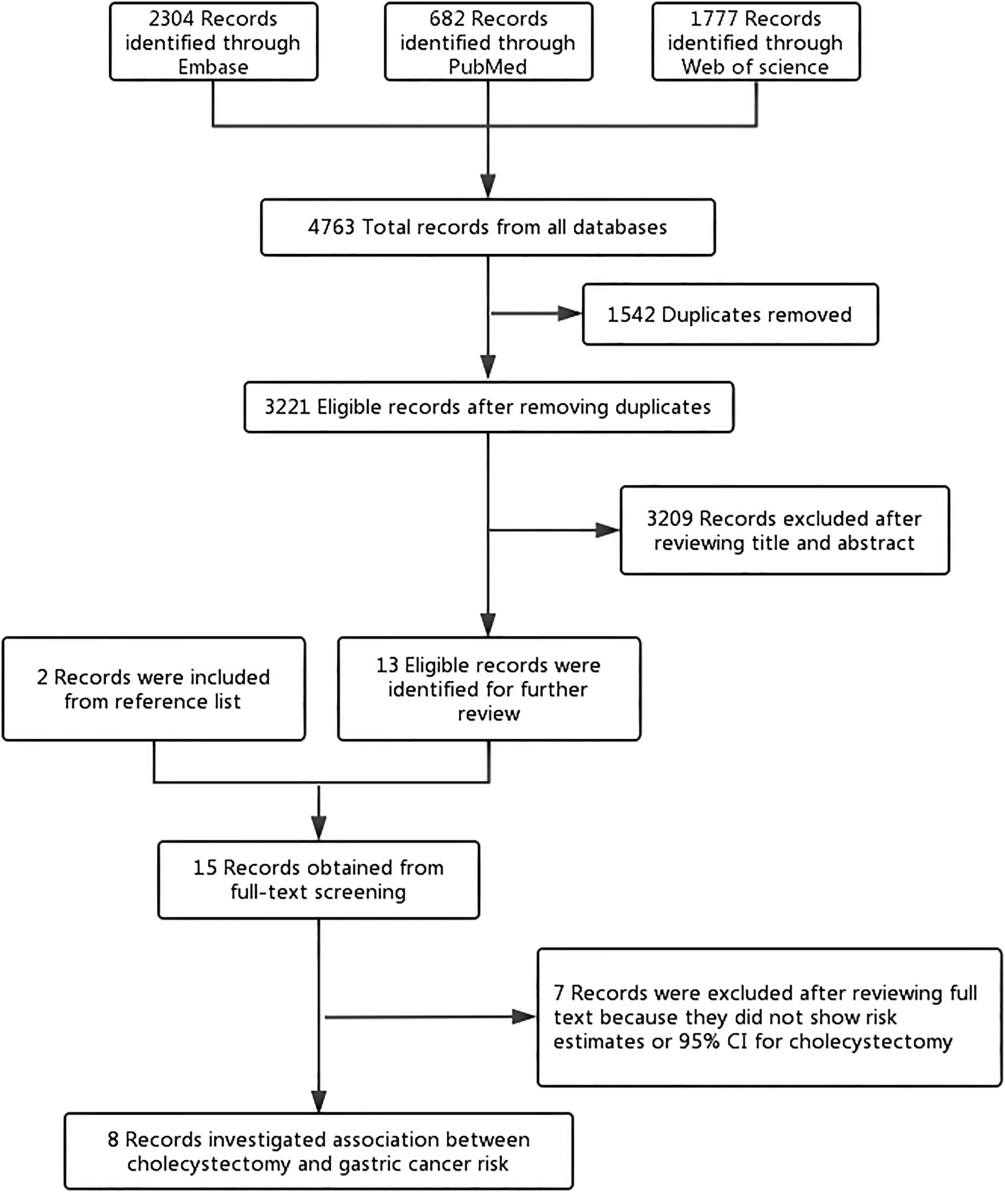
Flowchart of the study selection. The flowchart shows the process used to select studies for our meta-analyses focusing on the association between cholecystectomy and gastric cancer risk.

The search yielded 4,763 studies from three electronic biographic databases using a predefined search strategy. Two more studies (28, 29) were identified for full review by checking references. After removing 1,542 duplicates, 3,221 studies were screened based on title and abstract for further reading and 15 studies were eligible for further assessment by studying the full text. Eight studies met the inclusion and exclusion criteria and were selected for this systematic review and meta-analysis (**Figure 1**). The list of excluded studies was appended (**Supplementary Table S3**).

### Study Characteristics

The characteristics of the eight included studies are summarized in **Table 1**. These studies were published between 1984 and 2020 and included 26,063 GC patients with a range of 14–22,860 patients in individual studies. Of these eight studies, three (30, 32, 33) were case–control studies and five (14–17, 31) were cohort studies. Five (16, 17, 31–33) of these studies were conducted in Europe, two (14, 15) in Asia, and one (30) in the United States. Most studies collected the exposure through medical records or government databases. Furthermore, all studies required an objective GC diagnosis. All studies, except for one, were adjusted for age and sex (*n* = 6). Fewer studies were adjusted for the calendar year (*n* = 3). Specifically, one study was adjusted for more than five potential confounders in the primary analysis.

**Table 1:** Characteristics of studies included in the meta-analysis of the association between cholecystectomy and gastric cancer risk.

First author, ref, year	Location	Study design	No. of cases	No. of controls/cohorts	Exposure source	Risk estimate	Adjustments
Kim et al. (15), 2020	Korea	Cohort study	14	3,588	Medical record	SIR	N/A
Chen et al. (14), 2014	Taiwan	Cohort study	202	77,725	Database	HR	Age, sex, and comorbidities
Nogueira et al. (30), 2014	USA	Case-control study	22,860	100,000	Database	OR	Age, sex, and calendar year of selection, duration of Medicare benefits coverage
Fall et al. (16), 2007	Sweden	Cohort study	948	251,672	Database	SIR	Age, sex, and calendar year
Goldacre et al. (31), 2005	UK	Cohort study	15,31	374,067	Database	RR	Age, sex, calendar year, and district of residence
Freedman et al. (32), 2000	Sweden	Case-control study	2,62	820	Questionnaire	OR	Age, sex, tobacco use, alcohol use, body mass index, educational level, intake of fruit and vegetables, meal size, and physical activity
Sarli et al. (33), 1986	Italy	Case-control study	1,57	157	Medical record	OR	Age, sex, and geographic area of origin and dietary habits
Gustavsson et al. (17), 1984	Sweden	Cohort study	89	16,773	Database	RR	None

HR, hazard ratio; N/A, not available; OR, odds ratio; RR, relative risk; SIR, standardized incidence ratio.

### Risk of Bias Within Studies

**Supplementary Tables S4** and **S5** provide details of the study quality assessment as reflected by NOS scoring. Two studies were graded as high risk (15, 17). For the cohort study, three (15–17) studies that did not illustrate the source of cohort were not given a star for the selection of the unexposed cohort; two (15, 17) study failing to adjust any confounder was not given a star for comparability; two studies (14, 15) were not assigned a star for insufficient duration of follow-up. For the case–control study, one (33) study that included hospital-based controls was not given a star for the selection of control subjects; two (30, 33) case–control studies were not given a star for the definition of cases by the International Classification of Diseases code.

### RR of Cholecystectomy-Associated GC

**Figure 2:**
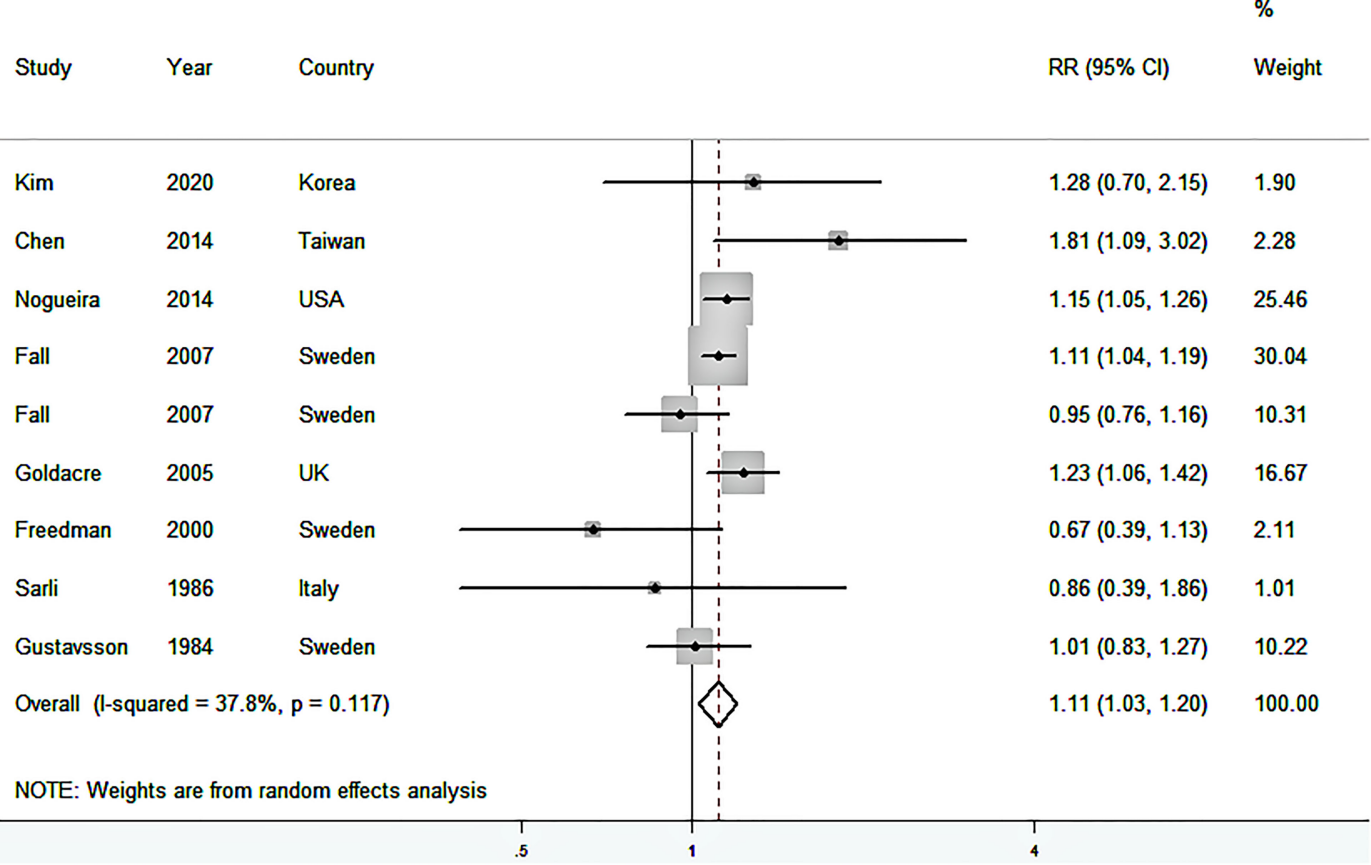
Forest plot (random-effects model) of the association between cholecystectomy and gastric cancer risk. Squares indicate study-specific relative risk (RR), where the size of the square reflects the study-specific statistical weight; horizontal lines indicate the 95% confidence interval (CI); diamonds denote the summary RR with 95% CI.

**Figure 2** shows the study-specific and summarized RRs and 95%CIs of GC for ever having cholecystectomy versus no history of cholecystectomy. Based on the eight studies, the summarized RR was 1.11 (95%CI: 1.03–1.20), with low heterogeneity among studies (*I* ^2^ = 37.8%). No publication bias was present (*P* for Begg’s test = 0.754, *P* for Egger’s test = 0.683) (**Supplementary Figure S1**).

The estimates by subgroups together with the results of the heterogeneity tests are given in **Table 2**. In subgroup analysis by study design, a significant result was observed after summarizing five cohort studies (14–17, 31) (RR = 1.12, 95%CI: 1.01–1.24), which was similar to the main finding, but not in case–control studies (30, 32, 33). In addition, similar situation was observed after summarizing six studies (14, 16, 30–33) with low risk (RR = 1.12, 95%CI: 1.02–1.23) instead of high risk (15, 17), two studies (14, 15) conducted in Asia (RR = 1.55, 95%CI: 1.06–2.26) instead of conducted in Europe (16, 17, 31–33), five studies (14, 16, 17, 30, 31) collecting exposure information on the basis of government database (RR = 1.12, 95%CI: 1.04–1.21) instead of other collecting exposure information methods (15, 32, 33), five studies (14, 16, 30–32) with more than 200 GC patients (RR = 1.12, 95%CI: 1.02–1.23) instead of less than 200 GC patients (15, 17, 33), six studies (14, 16, 30–33) adjusted for age or sex (RR = 1.12, 95%CI: 1.02–1.23), and three studies (16, 30, 31) adjusted for calendar year (RR = 1.13, 95%CI: 1.06–1.20). Of note, after summarizing these results of subgroup analyses by sex and anatomic subsite of GC in their primary analyses, significant results were only observed in male and non-cardia GC (**Table 2**).

**Table 2:** Risk estimates for cholecystectomy associated with gastric cancer in subgroup analysis.

	No. of studies	RR (95%CI)	*I* ^2^ (%)	*P* ^*^
**Region**				
Asia	2	1.55 (1.06–2.26)	0	0.370
Europe	5	1.07 (0.97–1.19)	41.2	0.131
USA	1	1.15 (1.05–1.26)	N/A	N/A
**Anatomic subsite of gastric cancer**				
Cardia	3	0.89 (0.78–1.02)	0	0.479
Non-cardia	2	1.17 (1.04–1.33)	74.1	0.050
**Number of cases**				
<200	3	1.03 (0.85–1.25)	0	0.666
≥200	5	1.12 (1.02–1.23)	55.6	0.047
**Study design**				
Cohort study	5	1.12 (1.01–1.24)	41.1	0.131
Case-control study	3	0.95 (0.66–1.38)	53.7	0.115
**Gender**				
Male	3	1.15 (1.00–1.32)	17.4	0.304
Female	3	0.99 (0.90–1.09)	0	0.827
**Risk of bias**				
Low risk	6	1.12 (1.02–1.23)	48.8	0.069
High risk	2	1.04 (0.85–1.27)	0	0.439
**Exposure collection**				
Database	5	1.12 (1.04–1.21)	41.8	0.126
Questionnaire	1	0.67 (0.39–1.14)	N/A	N/A
Medical record	2	1.04 (0.85–1.27)	0	0.439
**Adjustment for potential confounders**				
**Age**				
Yes	6	1.12 (1.02–1.23)	48.8	0.069
No	2	1.04 (0.85–1.27)	0	0.439
**Gender**				
Yes	6	1.12 (1.02–1.23)	48.8	0.069
No	2	1.04 (0.85-1.27)	0	0.439
**Calendar year**				
Yes	3	1.13 (1.06–1.20)	29.6	0.234
No	5	1.07 (0.79–1.45)	49.9	0.092

CI, confidence interval; N/A, not available; RR, relative risk.

*P-value for heterogeneity within each subgroup.

Additionally, no influential study was found in the sensitivity analyses, in which one study was omitted at a time and a summarized RR was calculated for the remainder of the studies. The estimated RR in this sensitivity analysis ranged from 1.09 (95%CI: 0.98–1.22, *I* ^2^ = 43.5%) to 1.13 (95%CI: 1.05–1.22, *I* ^2^ = 32.9%) (**Supplementary Figure S2**).

## Discussion

The current systematic review and meta-analyses included three case–control and five cohort studies involving 26,063 patients and 848,081 participants. The studies focused on cholecystectomy and GC risk. The findings revealed that cholecystectomy was associated with 11% increased risk of GC, with low heterogeneity among studies. This association was also significantly observed in cohort studies and studies with a low risk of bias. In subgroup analysis by anatomic subsite of GC, this effect was more pronounced in non-cardia GC compared with cardia GC. However, no evidence of the relationship between the duration of the follow-up period after cholecystectomy and GC risk was found.

One previous meta-analysis of observational studies was reported, but with inconsistent findings. In 2012, based on a meta-analysis of two case–control and three cohort studies, with 2,073 GC patients, Ge et al. (13) observed a non-significant excess risk of GC related to prior cholecystectomy (RR = 1.03, 95%CI: 0.93–1.13). Moreover, they also found a null association between cholecystectomy and risk of gastric cardia cancer (RR = 0.87, 95%CI: 0.65–1.17). However, the study had some limitations. First, the methodological quality of the included studies was not evaluated, and only a few subgroup analyses were made. Second, generalization of the results of previous meta-analysis in other countries was difficult because the included studies were conducted only in Western countries.

In the subgroup analysis layered by anatomic subsite of GC, we found that the positive association between cholecystectomy and non-cardia of GC risk. However, due to limited studies included in this subgroup analysis (n = 2), the probability of chance findings could not be ruled out. Additionally, although the two included studies both supported the aforementioned positive correlation results, Fall et al. (16) conducted the cohort study which had the limitations of small sample size and fewer confounding factors adjustment. Therefore, further studies are needed to explore the relationship between cholecystectomy and non-cardia of GC risk.

The underlying exact mechanisms of these contradictory links between cholecystectomy and GC risk have been unclear; however, some potential plausible mechanisms have been proposed to explain these findings. After cholecystectomy, bile flow changes, increasing the bile exposure of the stomach, changing bile salts, and subsequently changing the levels of metabolic hormone (34). Increased bile flow can cause bile to return to the stomach and esophagus, increasing the risk of GC (17). In addition, the presence of bile could cause another type of inflammation known as reactive gastritis (35). Moreover, clinical and epidemiological evidence have supported the functional relationship between chronic inflammation and cancer (36, 37). Furthermore, evidence suggests that one of these bile acids might be a weak mutagen, causing DNA damage, inducing frequent apoptosis, and ultimately increasing cancer incidence (38, 39).

To our knowledge, the present study is the most comprehensive meta-analysis of cholecystectomy and GC risk so far. The strengths of this study include the following: First, this meta-analysis involved a large sample size (26,063 patients and 848,081 participants) to evaluate the effect of cholecystectomy and GC risk, which increased the statistical power to detect the association. Second, numerous subgroup analyses were performed to analyze the study characteristics that might affect results, and sensitivity analyses were further performed to explore the heterogeneity in this study. Third, this present meta-analysis had no publication bias and low heterogeneity, and most of the included studies had a low risk of bias. All these strengths make the results of this study more convincing.

However, the study also had several limitations. First, a significant excess risk of GC related to cholecystectomy was observed in cohort studies, but not in case–control studies. Furthermore, the subgroup analysis of case–control studies represented moderate heterogeneity, which was higher than the subgroup of cohort studies. The case–control studies were prone to generate selection and recall bias, and the quality of the cohort studies was inconsistent, which might explain the observed heterogeneity in the study. Second, the different exposure rates of cholecystectomy varied among the included studies, which might be an important issue. Most included studies were conducted in European countries, and only a few were conducted in other countries. The exposure rate of cholecystectomy was observed to be 20% of the total participants in Taiwan but 10% of that in the United Kingdom (14, 31). Third, potential confounders that were not adjusted in individual studies could not be controlled. Although eight studies were included, the number of studies in each subgroup analysis was relatively small, leading to the need for further verification of some subgroup analysis results. In addition, only subgroup analyses for region, age, sex, exposure collection, and calendar year were conducted. The positive association between cholecystectomy and risk of GC persisted when the analysis of studies that adjusted for these confounders was restricted. Further studies should also consider whether the important risk factors of GC, such as *H. pylori* infection (4) and diet intake, affected the association of cholecystectomy and GC risk (3, 5). Fourth, only three included studies evaluated the association between cholecystectomy and different anatomic sites of stomach; therefore, inconsistent results were obtained for different anatomic sites with high heterogeneity. As cardia GC and non-cardia GC could have differences in the possibility of exposure to reflux bile, and data for further analysis were lacking, more studies are warranted to better elucidate this issue in the future. Fifthly, the cohort studies included in our meta-analysis differed in the evaluation of follow-up time, so it is difficult to summarize the RR and 95%CI of comparing longest vs shortest duration of the follow-up period. Future large cohort studies with longer follow-up time need to further explore the effect of cholecystectomy on gastric cancer risk. Additionally, the reasons for cholecystectomy in these included original studies were different or unknown. For example, Fall et al. identified participants through the Swedish National Inpatient Register, who had undergone cholecystectomy without illustrating the reason for the cholecystectomy (16). The patients who underwent cholecystectomy for symptomatic cholelithiasis or its complications, gallbladder polyp, or acalculous cholecystitis were included in the Kim et al. study (15). Therefore, further studies are needed to consider this in the future. Finally, only published studies were searched and included, while the gray literature and unpublished studies were ignored.

## Conclusions

The present systematic review and meta-analysis indicated that cholecystectomy had an increased risk of developing GC. Meanwhile, most subgroup analyses also supported the main findings. More large-scale prospective cohort studies are needed to validate these findings worldwide to gain further insights.

## Data Availability Statement

The data that support the findings of this study are available from the corresponding author upon reasonable request. Requests to access the datasets should be directed to yanli0227@126.com.

## Author Contributions

YL: conceived and designed the study. YY and M-HL: literature search, data curation and formal analysis, writing the original draft. All authors contributed to the article and approved the submitted version.

## Conflict of Interest

The authors declare that the research was conducted in the absence of any commercial or financial relationships that could be construed as a potential conflict of interest.

## Publisher’s Note

All claims expressed in this article are solely those of the authors and do not necessarily represent those of their affiliated organizations, or those of the publisher, the editors and the reviewers. Any product that may be evaluated in this article, or claim that may be made by its manufacturer, is not guaranteed or endorsed by the publisher.
