# Supplementary material for: Association Between Cholecystectomy and Gastric Cancer Risk: A Systematic Review and Meta-Analysis
Source: Front Oncol. 2022 Jan 31;12:667736. doi: 10.3389/fonc.2022.667736 (PMC8841561; doi:10.3389/fonc.2022.667736)
Supplement: Supplementary file 1 [file DataSheet_1.doc]

**Supplementary material**

**Supplementary Table S1.** PRISMA 2009 Checklist.

**Supplementary Table S2.** Search strategy used in the meta-analysis.

**Supplementary Table S3.** References of studies excluded in the meta-analysis.

**Supplementary Table S4.** Methodological quality of cohort studies included in the meta-analysis.

**Supplementary Table S5.** Methodological quality of case-control studies included in the meta-analysis.

**Supplementary Figure S1.** Funnel plot of publication biases of studies included in our meta-analysis focusing on the association between cholecystectomy and gastric cancer risk.

**Supplementary Figure S2.** Sensitivity analysis of studies included in the meta-analysis of the association between cholecystectomy and gastric cancer risk.

Supplementary Table S1. PRISMA 2009 Checklist.

| **Section/topic** | **#** | **Checklist item** | **Reported on page #** |
| --- | --- | --- | --- |
| **TITLE** | | |  |
| Title | 1 | Identify the report as a systematic review, meta-analysis, or both. | 1 |
| **ABSTRACT** | | |  |
| Structured summary | 2 | Provide a structured summary including, as applicable: background; objectives; data sources; study eligibility criteria, participants, and interventions; study appraisal and synthesis methods; results; limitations; conclusions and implications of key findings; systematic review registration number. | 2-3 |
| **INTRODUCTION** | | |  |
| Rationale | 3 | Describe the rationale for the review in the context of what is already known. | 4-5 |
| Objectives | 4 | Provide an explicit statement of questions being addressed with reference to participants, interventions, comparisons, outcomes, and study design (PICOS). | 4-5 |
| **METHODS** | | |  |
| Protocol and registration | 5 | Indicate if a review protocol exists, if and where it can be accessed (e.g., Web address), and, if available, provide registration information including registration number. | None |
| Eligibility criteria | 6 | Specify study characteristics (e.g., PICOS, length of follow-up) and report characteristics (e.g., years considered, language, publication status) used as criteria for eligibility, giving rationale. | 6 |
| Information sources | 7 | Describe all information sources (e.g., databases with dates of coverage, contact with study authors to identify additional studies) in the search and date last searched. | 5-6 |
| Search | 8 | Present full electronic search strategy for at least one database, including any limits used, such that it could be repeated. | 5-6  Supplementary Table S2 |
| Study selection | 9 | State the process for selecting studies (i.e., screening, eligibility, included in systematic review, and, if applicable, included in the meta-analysis). | 6 |
| Data collection process | 10 | Describe method of data extraction from reports (e.g., piloted forms, independently, in duplicate) and any processes for obtaining and confirming data from investigators. | 6-7 |
| Data items | 11 | List and define all variables for which data were sought (e.g., PICOS, funding sources) and any assumptions and simplifications made. | 6-7 |
| Risk of bias in individual studies | 12 | Describe methods used for assessing risk of bias of individual studies (including specification of whether this was done at the study or outcome level), and how this information is to be used in any data synthesis. | 7-8 |
| Summary measures | 13 | State the principal summary measures (e.g., risk ratio, difference in means). | 7-8 |
| Synthesis of results | 14 | Describe the methods of handling data and combining results of studies, if done, including measures of consistency (e.g., I^2^) for each meta-analysis. | 7-8 |

| **Section/topic** | **#** | **Checklist item** | **Reported on page #** |
| --- | --- | --- | --- |
| Risk of bias across studies | 15 | Specify any assessment of risk of bias that may affect the cumulative evidence (e.g., publication bias, selective reporting within studies). | 6-8 |
| Additional analyses | 16 | Describe methods of additional analyses (e.g., sensitivity or subgroup analyses, meta-regression), if done, indicating which were pre-specified. | 6-8 |
| **RESULTS** | | |  |
| Study selection | 17 | Give numbers of studies screened, assessed for eligibility, and included in the review, with reasons for exclusions at each stage, ideally with a flow diagram. | 8  Figure 1  Supplementary Table S3 |
| Study characteristics | 18 | For each study, present characteristics for which data were extracted (e.g., study size, PICOS, follow-up period) and provide the citations. | 8-9  Table 1 |
| Risk of bias within studies | 19 | Present data on risk of bias of each study and, if available, any outcome level assessment (see item 12). | 9  Supplementary Tables S4 and S5 |
| Results of individual studies | 20 | For all outcomes considered (benefits or harms), present, for each study: (a) simple summary data for each intervention group (b) effect estimates and confidence intervals, ideally with a forest plot. | 9-10  Table 1 |
| Synthesis of results | 21 | Present results of each meta-analysis done, including confidence intervals and measures of consistency. | 10-11 |
| Risk of bias across studies | 22 | Present results of any assessment of risk of bias across studies (see Item 15). | 10-11  Table2 |
| Additional analysis | 23 | Give results of additional analyses, if done (e.g., sensitivity or subgroup analyses, meta-regression [see Item 16]). | 11 |
| **DISCUSSION** | | |  |
| Summary of evidence | 24 | Summarize the main findings including the strength of evidence for each main outcome; consider their relevance to key groups (e.g., healthcare providers, users, and policy makers). | 11-15 |
| Limitations | 25 | Discuss limitations at study and outcome level (e.g., risk of bias), and at review-level (e.g., incomplete retrieval of identified research, reporting bias). | 14-15 |
| Conclusions | 26 | Provide a general interpretation of the results in the context of other evidence, and implications for future research. | 15 |
| **FUNDING** | | |  |
| Funding | 27 | Describe sources of funding for the systematic review and other support (e.g., supply of data); role of funders for the systematic review. | None |
|  |  |  |  |

*From:*  Moher D, Liberati A, Tetzlaff J, Altman DG, The PRISMA Group (2009). Preferred Reporting Items for Systematic Reviews and Meta-Analyses: The PRISMA Statement. PLoS Med 6(6): e1000097. doi:10.1371/journal.pmed100009

**Supplementary table S2. Search strategy used in the meta-analysis**

| **Literature search strategy in Medline (PubMed)** |
| --- |
| 1: cholecystectomy OR laparoscopic cholecystectomies OR celioscopic cholecystectomies OR cholelithiasis OR cholecystolithiasis OR choledocholithiasis OR gallstones |
| 2: gastric OR stomach |
| 3: cancer OR tumor OR carcinoma OR neoplasm |
| 4: 2 AND 3 |
| 5: 1 AND 4 (n = 682) |
| **Literature search strategy in Embase** |
| 1: cholecystectomy OR laparoscopic cholecystectomies OR celioscopic cholecystectomies OR cholelithiasis OR cholecystolithiasis OR choledocholithiasis OR gallstones |
| 2: gastric OR stomach |
| 3: cancer OR tumor OR carcinoma OR neoplasm |
| 4: 2 AND 3 |
| 5: 1 AND 4 (n = 2304) |
| **Literature search strategy in Web of Science** |
| 1: Search TS = (cholecystectomy OR laparoscopic cholecystectomies OR celioscopic cholecystectomies OR cholelithiasis OR cholecystolithiasis OR choledocholithiasis OR gallstones) |
| 2: Search TS = (gastric OR stomach) |
| 3: Search TS = (cancer OR tumor OR carcinoma OR neoplasm) |
| 4: 2 AND 3 |
| 5: 1 AND 4 (n = 1777) |

**Supplementary Table S3. References of studies excluded in the meta-analysis.**

| **Not show risk estimates or 95% confidence interval for association between cholecystectomy and gastric cancer risk (n = 7)** |
| --- |
| 1. Zhao X, Wang N, Sun Y, et al. Screen-detected gallstone disease and risk of liver and pancreatic cancer: The Kailuan Cohort Study[J]. Liver Int, 2020,40(7):1744-1755. |
| 2. Kang S H, Kim Y H, Roh Y H, et al. Gallstone, cholecystectomy and risk of gastric cancer[J]. Ann Hepatobiliary Pancreat Surg, 2017,21(3):131-137. |
| 3. Shabanzadeh D M, Sorensen L T, Jorgensen T. Association Between Screen-Detected Gallstone Disease and Cancer in a Cohort Study[J]. Gastroenterology, 2017,152(8):1965-1974. |
| 4. Tavani A, Rosato V, Di Palma F, et al. History of cholelithiasis and cancer risk in a network of case-control studies[J]. Ann Oncol, 2012,23(8):2173-2178. |
| 5. Johansen C, Chow W H, Jorgensen T, et al. Risk of colorectal cancer and other cancers in patients with gall stones[J]. Gut, 1996,39(3):439-443. |
| 6. Maringhini A, Moreau J A, Melton L R, et al. Gallstones, gallbladder cancer, and other gastrointestinal malignancies. An epidemiologic study in Rochester, Minnesota[J]. Ann Intern Med, 1987,107(1):30-35. |
| 7. Ichimiya H, Kono S, Ikeda M, et al. Cancer mortality among patients undergoing cholecystectomy for benign biliary diseases[J]. Jpn J Cancer Res, 1986,77(6):579-583. |

**Supplementary Table S4. Methodological quality of cohort studies included in the meta-analysis**

| **First author [ref], year** | **Selection** | | | | **Comparability** | **Outcome** | | | **Risk of bia**s **^d^** |
| --- | --- | --- | --- | --- | --- | --- | --- | --- | --- |
|  | Representativeness of the exposed cohort | Selection of the unexposed cohort | Ascertainment of exposure | Outcome of interest not present at start of study | Control for  important factor or additional factor ^a^ | Assessment of outcome | Follow-up  long enough for outcomes to occur ^b^ | Adequacy of follow-up of cohorts ^c^ |  |
| Kim et al,^15^, 2020 | * | **-** | * | * | - | * | **-** | * | High risk |
| Chen et al,^14^,2014 | * | * | * | * | ** | * | **-** | * | Low risk |
| Fall et al,^16^,2007 | * | **-** | * | * | ** | * | * | * | Low risk |
| Goldacre et al,^38^,2005 | * | * | * | * | ** | * | * | * | Low risk |
| Gustavsson et al,^17^,1984 | * | **-** | * | * | - | * | * | * | High risk |

^a^ A maximum of 2 stars could be awarded for this item. Studies that controlled for age received one star, whereas studies that controlled for other important confounders such as sex received an additional star.

^b^ A cohort study with a follow-up time >10 years was assigned one star.

^c^ A cohort study with a follow-up rate >75% was assigned one star.

^d^ Studies that obtained a full scores at least two domains were considered to have a low risk of bias, other situations were considered as high risk.

**Supplementary Table S5. Methodological quality of case-control studies included in the meta-analysis**

| **First author [ref], year** | **Selection** | | | | **Comparability** | **Exposure** | | | **Risk of bia**s **^c^** |
| --- | --- | --- | --- | --- | --- | --- | --- | --- | --- |
|  | Adequate definition of cases | Representativeness of cases | Selection of control subjects | Definition of control subjects | Control for important factor or additional factor ^a^ | Exposure assessment | Same method of ascertainment for all subjects | Non response Rate ^b^ |  |
| Nogueira et al,^35^,2014 | **-** | * | * | * | ** | * | * | * | Low risk |
| Freedman et al,^36^,2000 | * | * | * | * | ** | * | * | * | Low risk |
| Sarli et al,^37^,1986 | **-** | * | **-** | * | ** | * | * | * | Low risk |

^a^ A maximum of 2 stars could be awarded for this item. Studies that controlled for age received one star, whereas studies that controlled for other important confounders such as sex received an additional star.

^b^ One star was assigned if there was no significant difference in the response rate between control subjects and cases by using the chi-square test (P>0.05).

^c^ Studies that obtained a full scores at least two domains were considered to have a low risk of bias, other situations were considered as high risk.


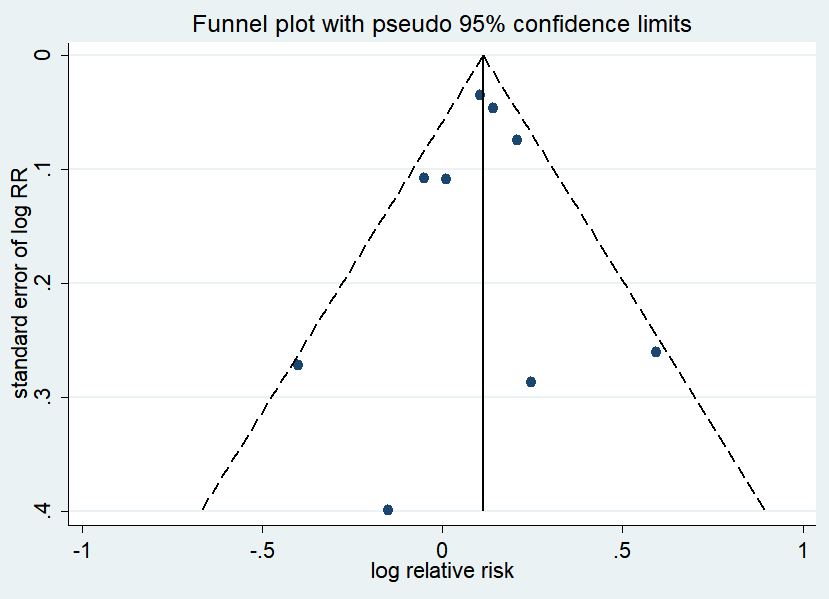


**Fig. S1. Funnel plot of publication biases of studies included in our meta-analysis focusing on the association between cholecystectomy and gastric cancer risk. Each dot represents an individual study.**


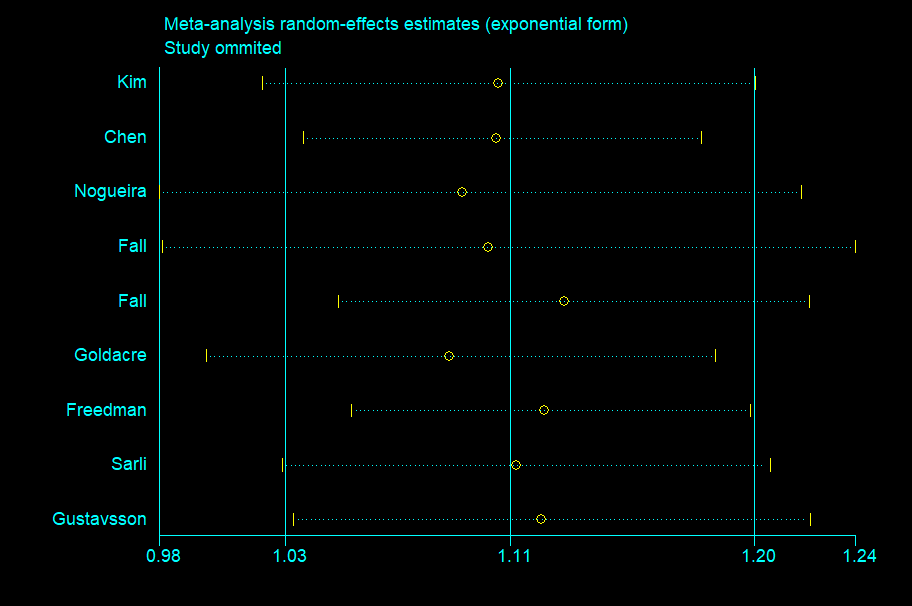


**Fig. S2. Sensitivity analysis of studies included in the meta-analysis of the association between cholecystectomy and gastric cancer risk.**
